# Rewriting the transcriptome: adenosine-to-inosine RNA editing by ADARs

**DOI:** 10.1186/s13059-017-1347-3

**Published:** 2017-10-30

**Authors:** Carl R. Walkley, Jin Billy Li

**Affiliations:** 1 St Vincent’s Institute of Medical Research, Fitzroy, Victoria 3065 Australia; 2 Department of Medicine, St Vincent’s Hospital, University of Melbourne, Fitzroy, Victoria 3065 Australia; 3 Department of Genetics, Stanford University, Stanford, CA 94305 USA

## Abstract

One of the most prevalent forms of post-transcritpional RNA modification is the conversion of adenosine nucleosides to inosine (A-to-I), mediated by the ADAR family of enzymes. The functional requirement and regulatory landscape for the majority of A-to-I editing events are, at present, uncertain. Recent studies have identified key in vivo functions of ADAR enzymes, informing our understanding of the biological importance of A-to-I editing. Large-scale studies have revealed how editing is regulated both in *cis* and in *trans*. This review will explore these recent studies and how they broaden our understanding of the functions and regulation of ADAR-mediated RNA editing.

## Introduction

The post-transcriptional modification of RNA is a key process controlling the output of the genome, shaping the transcriptional landscape and ultimately cellular and organismal fate. Many types of RNA regulation have been identified, from differential splicing and isoform usage through to disctinct classes of chemical modification [1]. There are greater than 100 known distinct modifications that can occur on and to RNA, highlighting the higher order regulation that can be layered onto RNA [2]. Of the modifications described to date, a highly pervasive and prevalent form is the direct enzymatic deamination of adenosine nucleosides in RNA, resulting in their conversion to inosine, a process termed A-to-I editing [3–5].

A-to-I editing was initially identified as an activity causing the unwinding of transfected RNA duplexes in *Xenopus* eggs [6, 7]. It was subsequently identified that this unwinding activity was the result of the covalent modification of the RNA, and that the activity was specific to double-stranded RNA (dsRNA) [8, 9]. This activity was found in a range of species, including mammals. The specific characteristic of this modification was identified based on the analysis of the sequence differences between the genomic DNA and mRNA sequences of the GluA2 glutamate receptor (gene name *Gria2*) [10]. In this example, there was a change in transcript and protein sequence from that predicted by the genomic DNA, with an arginine codon (CGG) in the mRNA in place of the genomically encoded glutamine codon (CAG). Inosine is resolved as guanosine upon sequencing and also by the translational machinery, meaning that A-to-I editing is identified as A-to-G variations in the sequence traces (either Sanger or RNA-seq) compared with the genomic sequence [10–14].

A-to-I editing is performed by the adenosine deaminase acting on RNA (ADAR) family of proteins [15–18]. ADARs catalyze the deamination of adenosine to inosine, through the hydrolytic deamination of the 6-position of adenosine [19]. Inosine preferentially base pairs with cytidine. The editing of adenosines can result in a decrease or an increase in base pairing of the dsRNA substrate depending upon the sequence context. While conceptually the identification of an edited adenosine should be relatively straightforward by comparing the transcript sequence to the genome, this has not proven to be the case [20–24]. Several factors need to be considered to accurately define A-to-I editing: that editing occurs generally at low frequency (the majority of editing occurs at less than 20% frequency); that errors can be introduced by random hexamers used to generate the samples and by the sequencing technology; and that paralogs and closely related sequences (including SNPs) need to be able to be distinguished so that the events can be assigned accurately within the genome [21]. The later issue becomes more relevant when editing of repeat regions, such as *Alu* and retrotransposons, is assessed due to the high level of sequence similarity present in these regions.

Our knowledge of editing was largely confined to a select few well-studied targets, until the parallel advances in computational methods and sequencing approaches that generate significantly greater transcriptome coverage converged to allow the accurate identification of editing in many different species. Since the identification of this modification and with the relatively recent advances in sequencing methods, the number of known sites that can be subjected to A-to-I editing has grown exponentially, with current estimates of up to 100 million possible editing events in the human genome [25, 26].

## Types of editing

Two primary types of A-to-I editing have been defined. The first is site-selective editing [25, 27–29]. This type of editing refers to the deamination of a specific adenosine in an RNA [10]. This can occur in isolation with no editing detected at neighboring adenosines or in short clustered regions within a given transcript (see [30] for an example). The efficiency of site-selective editing of a given base varies widely, from near 100% for the canonical example of *Gria2* to less than 0.1%, with the majority of editing occurring at a frequency of less than 20% when assessed genome-wide [31]. An adenosine subjected to editing in one tissue or subregion of a tissue may be differentially edited in other tissues or regions of the same tissue, suggesting that regulation of editing occurs and that it does not represent an “all or none” phenomenon [31]. There are many additional examples of highly specific A-to-I editing events [10, 14, 32–34]. Site-selective editing is best associated with transcript recoding, where the editing causes a change in the protein sequence and subsequent function [14]. Despite the capacity for protein recoding arising from A-to-I editing, the proportion of editing events that result in this outcome are a very small minority of those now described in mammalian genomes, and the degree of conservation of these is generally low [26, 35]. The consequences of recoding can vary, from the introduction of silent mutations with no discernable consequence for protein function through to mutations that alter the function of the protein dramatically, with the GluA2 Q/R site defining this latter paradigm [12–14, 32, 33].

The second and distinctive type of A-to-I editing is hyper-editing [36, 37], which refers to a similar phenomenon as editing enriched regions (EERs) [38, 39]. Hyper-editing is indicated by the editing of a large or excessive proportion of adenosines in close proximity to each other within the same transcript [40–42]. In mammals, this class of editing is mostly associated with regions of repetitive sequence where high levels of homology arise from the base pairing of inverted repeats, resulting in the editing of a high proportion of adenosines in a short region of several hundred base pairs [36]. This primarily occurs in intronic regions and 3′ UTRs in the mammalian context. In humans and primates this includes *Alu* elements and other types of repetitive regions [26, 28, 35, 43]. This type of extensive editing has also been observed in viral sequences, where the viral dsRNA can be subjected to extensive editing in the infected cell [9, 44–47].

## Expansion of RNA editing sites

The initial identification of A-to-I editing sites was largely based on serendipitous discoveries stemming from the detailed assessment of a single transcript [10, 48]. Evidence for hyper-editing first arose from virology, where it was noted that the dsRNA of certain types of virus could be heavily modified [9, 49]. Methods were developed, and more recently adapted for use with high-throughput sequencing, to allow identification of inosine-containing transcripts. These approaches rely on either the preferential cleavage of inosine-containing transcripts by enzymes such as RNAse T1, or upon the chemical conversion of inosine by cyanoethylation, to allow identification of edited sites [50–52].

Methodologies to systematically map A-to-I editing have primarily utilized the in silico analysis of expressed sequence tag databases or, more recently, the analysis of large RNA-seq datasets [26–28, 31, 35, 43, 53–55]. With the advent of high-throughput sequencing technologies, which have enabled base resolution analysis of most of the genome and the rapid cost per base reductions in their utilization, the numbers of editing sites catalogued has dramatically expanded [25, 26, 35, 56]. Targetted approaches, such as microfluidic multiplex PCR and sequencing (mmPCR-seq), allowing the highly accurate sampling of editing at a significant number of known editing sites across a range of tissues/samples at low cost, have added significantly to our ability to profile editing across tissues of an organism [57]. These approaches have also made possible the comparison of editing among species and phyla, providing important understanding of its prevalence and clues to its function in different contexts [42, 58]. The analysis of editing across and within species has been highly informative to our understanding of the extent and consequences of A-to-I editing over evolution [56, 59–61]. The inclusion of genetically modified cells and organisms, such as tissues from the various ADAR knockout animals and cell lines with reduced ADAR expression/function, has enabled the experimental validation of large numbers of the sites that have been identified computationally in addition to the discovery of additional sites [40, 41, 62, 63]. These complementary approaches have provided important validation of the methods and have been extended to begin to understand the differential effects and site preferences of the individual ADAR proteins.

Intuitively, RNA editing sites can be identified by finding genetic variants (A-to-G transitions on the forward strand, T-to-C on the reverse strand) present in the RNA-seq data but absent in the matched whole genome sequence from the same individual or species [28, 64]. More recently, methods have evolved and a number of rigorous methods have been established to identify RNA editing sites, including those that can use RNA-seq alone rather than a reference genome [29, 65, 66]. Furthermore, special techniques have been developed to identify hyper-editing sites that often escape from the conventional approaches [36]. This has been necessary due to the excessive numbers of edited bases in regions of hyper-editing which can impact on genomic alignment of these regions, making differentiation of these regions from sequencing errors of “bad reads” imperative. A historical view of the development of methods to reliably identify RNA editing sites is summarized in detail in a recent review (see reference [26] for a detailed perspective on this topic). Several databases are publically available to assess and query RNA-editing sites across species, including RADAR [35], DARNED [67, 68], and REDIbd/REDItools [69].

## ADAR proteins

The numbers and conservation of ADARs varies across species. Mammals have three proteins: ADAR1 (*ADAR*), ADAR2 (*ADARB1*), and ADAR3 (*ADARB2*); *Drosophila melanogaster* has a single *Adar* (phenotypically most similar to mammalian ADAR2 [70, 71]); and *Caenorhabditis elegans* has two genes, *adr-1* and *adr-2* (phenotypically most similar to ADAR3 and ADAR2, respectively [72]). Each ADAR has dsRNA binding regions and a highly conserved carboxy terminal catalytic domain, distantly related to the bacterial cytidine deaminases [17, 73]. Mammalian ADAR1 and ADAR2 have demonstrated catalytic activity and participate in A-to-I editing; in contrast, no editing activity has been detected with ADAR3 on known subtrates and it appears to be catalytically inactive [74, 75]. Unlike ADAR1 and ADAR2, ADAR3 does not appear to homodimerize and this may be an important contributor to its lack of activity [17, 74]. Similarly, in *C. elegans adr-2* is capable of A-to-I editing while *adr-1*, akin to mammalian ADAR3, does not display editing activity [72].

The expression of each of the ADARs varies across development and tissues in mammals [76]. ADAR1 is widely expressed throughout the body and is the most highly expressed ADAR outside the central nervous system (CNS). A unique feature of ADAR1 is that it can be expressed as two distinct editing competent isoforms, and increasing evidence supports that these may have both overlapping and distinctive functions [18, 30, 77, 78]. ADAR1 is expressed as a consititutive p110 kDa isoform (ADAR1 p110), which localizes primarily to the nucleus, and an inducibile ADAR1 p150 isoform [79]. The larger isoform can be induced by activation of the interferon and innate immune sensing system and localizes to the cytoplasm [18]. ADAR2 and ADAR3 are most highly expressed in the brain and CNS, with expression more restricted in other tissues. ADAR2 contributes significantly to editing in the testis in the mouse [80]. The completion of detailed body maps and single cell studies of gene expression will enable a significantly refined understanding of when and how different ADARs are expressed throughout the body.

The phenotypes associated with loss of function of ADARs differ between species. In *C. elegans* deletion of *adr-1* or *adr-2* resulted in defects in chemotaxis [81], phenotypes that are consistent with a role in neuronal function. Interestingly, the chemotaxis defect could be rescued by concurrent deletion of components of the RNAi pathway, including *rde-1* and *rde-4*, implicating an interaction between RNA editing and RNAi pathways [82]. Very recently, the chemotactic defect in *adr-2*-deficient *C. elegans* has been determined to be an editing-dependent effect [83]. The normal expression of the mRNA of *clec-41*, a predicted C-type lectin protein, was dependent upon editing by ADR-2. In ADR-2-deficient cells, the expression of *clec-41* was significantly reduced. When *clec-41* expression was restored in *adr-2*-deficient neural cells, the chemotactic defect could be rescued, providing direct evidence that neuronal/chemotactic phenotypes of *adr-2* mutants can be attributed to altered gene expression of an edited transcript [83].

Deletion of the single ADAR in *Drosophila* resulted in behavioral and locomotion abnormalities with brain lesions upon aging [70, 84, 85]. More recently, hypomorphic alleles have been established in *Drosophila* which have defects in sleep patterns [86], with evidence for a conserved disruption of circadian rhythm in *Adar2* ^*–/–-*^ mice [87]. In both *C. elegans* and *Drosophila*, the germline deletion of ADARs is compatibile with life and the mutants are viable but phenotypic [88]. Phylogenetic analysis demonstrated that mammalian ADAR2 could rescue *Drosophila* Adar null mutants, but that mammalian ADAR1 could not [71]. This result, coupled with evolutionary analysis, suggested that ADAR1 and ADAR2 evolved separately and have conserved, but specialized, functions. Analysis of mammalian mutant models has now confirmed this.

In mice, deletion of *Adar2* resulted in the fully penetrant development of postnatal seizures that ultimately result in death by 20–25 days of age [13]. This phenotype was rescued by the substitution of a single adenine to guanine in the Q/R position of the *Gria2* gene, mimicking constitutive editing at this site [11, 13]. The rescued *Adar2* ^–/–^ *Gria2* ^*R/R*^ animals have a normal lifespan, are fertile, but have some subtle phenotypes that were revealed by broad-based phenotyping [89]. This elegant model of rescue of lethality by a single A-to-I site substitution within a single RNA substrate illustrated definitively the paradigm of ADAR-mediated editing resulting in protein recoding as an essential consequence of A-to-I editing. Retrospectively, this result was also confounding as it suggested that editing of a large range of sites that have been subsequently defined was of limited biological relevance. Alternatively, it hinted that most editing may be required for “fine tuning” rather than being essential for homeostasis in mammals, and so may require specific contexts or settings for phenotypes to be revealed. However, as we now appreciate, the levels of redundancy and overlap of editing substrates between ADAR1 and ADAR2 are important considerations when interpreting the in vivo results.

In contrast to the *Adar2* ^*–/–-*^ phenotype, the deletion of Adar1 (*Adar1* ^*–/–*^, both p110 and p150 isoforms [30, 90]), the deletion of the p150 isoform specifically (*Adar1p150* ^*–/–-*^ [77]), or the specific inactivation of the editing activity/catalytic domain (*Adar1* ^*E861A/E861A*^, both p110 and p150 are editing deficient [41]) resulted in embryonic lethality between E11.5 and E13.5. These animals are characterized by a failure in fetal hematopoiesis and liver disintegration, marked by high levels of cell death. Subsequent studies identified the profound deregulation of transcripts related to the innate immune sensing (interferon) response upon deletion or mutation of ADAR1 [91]. Using genetic intercrosses of the *Adar1* mutants it has been identified by several groups including our own that a key in vivo function of ADAR1 is to modify endogenous RNA, via editing, to prevent activation of the cytosolic dsRNA sensing pathway centred on MDA5 and its downstream effector MAVS (Table 1) [41, 78, 92]. A number of genetic pathways have been tested by crossing to the *Adar1* mutants and assessing rescue of viability. Of the pathways tested in vivo, to date the only significant rescue has been achieved with the deletion of MDA5 and MAVS [41, 78, 92]. This function is unique to ADAR1, and is not shared by other mammalian ADARs. It was recently reported in human cell lines that deletion of RNaseL could rescue the viability of *ADAR1* ^*–/–*^ cell lines, in a comparable manner to deletion of MAVS [93]. It is not presently clear whether the requirement for RNaseL is downstream of MDA5/MAVS signaling or can be initiated independently of this axis and whether the effect is physiologically relevant in vivo.

**Table 1:** Summary of the different murine crosses performed to identify rescue of the *Adar1* and *Adar2* murine phenotypes, respectively

*Adar* allele	Genetic modifier (gene/protein)	Method	Function/substrate	Outcome at birth	Reference(s)
*Adar1* ^*–/–*^ (p110 and p150				E11.5–12.0 lethal	[30, 90]
	*Ifih1* ^*–/–*^ (MDA5)	Mouse cross	Long paired dsRNA	Rescue: majority die by 2 days old	[78]
	*Mavs* ^*–/–*^ (MAVS)	Mouse cross; Crispr cell line	Effector of RIG-I and MDA5	Rescue: majority die by 2 days old; small number survive up to 20 days	[78, 92, 93]
	*Rnasel* ^*–/–*^ (RNase L)	Crispr cell line	Endoribonuclease; cleaves dsRNA	Rescue (cell lines)	[93]
	*Eif2ak2* ^*–/–*^ (PKR)	Mouse cross	dsRNA-activated serine/threonine kinase	No rescue	[90]
	*Tmem173* ^*–/–*^ (STING)	Mouse cross	Cytosolic DNA sensor	No rescue	[78]
	*Ddx58* ^*–/–*^ (RIG-I)	Mouse cross	Short 5′ phos RNA (ds and ss)	No rescue	[78]
	*Stat1* ^*–/–*^ (STAT1)	Mouse cross	Transcriptional effector of interferon pathway	No rescue; lethal by E15.5	[92]
	*Ifnar* ^*–/–*^ (IFNRα)	Mouse cross	Type I interferon receptor	No rescue; lethal at E14.5–15.5	[78, 92]
	*Ifnar* ^*–/–*^ *Ifngr* ^*–/–*^ (IFNαR/IFNγR)	Mouse cross	Type I and II interferon receptor	No rescue; lethal at E15.5	[40]
	*Trp53* ^*–/–*^ (p53)	Mouse cross	Tumor suppressor; can modify cell death	No rescue	Unpublished (J. Hartner and C. Walkley)
*Adar1p150* ^*–/–*^ (p150 KO only)				E11.5–12.0 lethal	[77]
	*Mavs* ^*–/–*^ (MAVS)	Mouse cross	Effector of RIG-I and MDA5	Rescue: majority survive to 20 days	[78]
*Adar1E861A/E861A* (editing deficient)				E13.5 lethal	[41]
	*Ifih1* ^*–/–*^ (MDA5)	Mouse cross; cell lines	Long paired dsRNA	Rescue: majority survive normally In vitro cell lines—rescue	[41, 106]
	*Ifnar* ^*–/–*^ *Ifngr* ^*–/–*^ (IFNαR/IFNγR)	Mouse cross	Type I and II interferon receptor	No rescue; lethal at E15.5	Unpublished (B. Liddicoat and C. Walkley)
*Adar2* ^*–/–*^				Early post-natal lethal (~20 days); seizures	[13]
	*Gria2 R/R* (GluA2 R/R)	Mouse cross	GluA2 glutamate receptor	Rescue; adults normal	[13, 89]

A question that has not been definitively resolved is the extent to which the phenotypes seen in the different mutant mouse models are due to editing-dependent or editing-independent functions. This is reasonably clear for the *Adar2* ^*–/–*^ animals, with the profound rescue of the phenotype in these mice by the *Gria2* ^*R/R*^ allele demonstrating that the physiologically most important function of ADAR2 is A-to-I editing. The *Adar2* ^*–/–*^ *Gria2* ^*R/R*^ animals do have additional subtle phenotypes that were revealed after a comprehensive phenotypic analysis and testing suggesting that there are specific requirements for ADAR2 outside of *Gria2* editing; however, whether these reflect the lack of editing of specific substrates or editing-independent functions is not clear [89]. In the case of ADAR1, a number of editing-independent functions have been proposed and phenotypes observed in rescued mice that were interpreted as independent of the editing activity of ADAR1. These range from roles in miRNA biogenesis [94–100], affecting mRNA stability [100–102], alternative 3′ UTR usage [97], and altering RNA splicing [103, 104] and the rates and efficiency of translation [105]. In vivo, the small numbers of *Adar1* ^*–/–*^ *Mavs* ^*–/–*^ and *Adar1p150* ^*–/–*^ *Mavs* ^*–/–*^ rescued mice that survived past 10 days of age had developmental defects in the kidney, small intestine, and lymph node and a failure of B lymphopoiesis [78]. In contrast to these reported roles for editing-independent activities of ADAR1, we found that an Adar1 editing-deficient allele (*Adar1* ^*E861A*^) demonstrated highly comparable phenotypes in both a germline-deficient or acute adult somatic deletion model to ADAR1 null alleles [40, 41, 106]. That the specific absence of editing, with a protein still being expressed, and the complete absence of the protein are so similar argues strongly that there are limited additional in vivo functions for the protein beyond editing. These genetic results do not exclude context-specific functions of ADAR1 independent of editing that were either not assessed or not active in the cell types assessed (primarily hematopoietic cells). At an organismal level A-to-I editing is the most essential function of ADAR1 and this function is required to prevent inappropriate activation of the innate immune system by endogenous RNA species.

No editing activity has been demonstrated by ADAR3. The role of mammalian ADAR3 is less clear, but data are accumulating from both *C. elegans* and mammalian models that ADAR3 may act to reduce the availability of substrates for ADAR1 or ADAR2, resulting in a net overall inhibitory effect on editing levels [72, 75, 76]. No phenotypes similar to those identified in the *Adar1* and *Adar2* mutants have been reported for *Adar3* ^*–/–*^ animals to date. Therefore, it is the combination of expression patterns of the different ADAR isoforms that can determine the nature and extent of editing in a given cell and tissue, with ADAR3 providing a counterpoint to ADAR1 and ADAR2 [76].

**Fig. 1:**
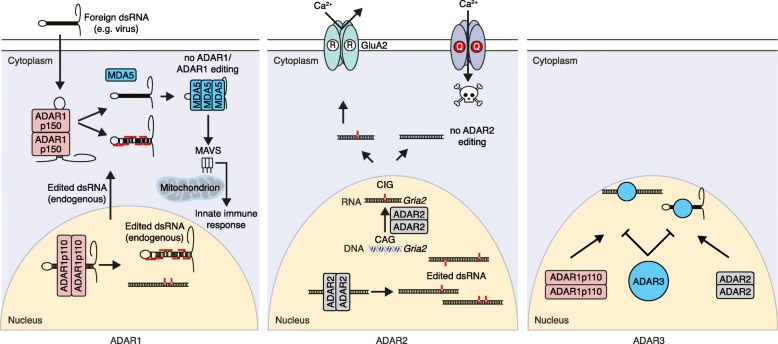
The roles of ADAR1, ADAR2, and ADAR3. ADAR1 is present in the nucleus (ADAR1 p110) and cytoplasm (ADAR1 p150) and can edit endogenous RNA. ADAR1 is required to edit endogenous RNA to prevent the activation of the cytosolic pattern recognition receptor MDA5 in the cytosol, leading to induction of the innate immune/interferon response. ADAR1 can also edit viral dsRNA and participate in the innate immune response as a direct interferon-stimulated gene (*ADAR1* p150 isoform). The absence of ADAR1 or the absence of ADAR1-mediated editing leads to innapropriate activation of the MDA5–MAVS axis. ADAR2 is essential for site-selective editing and is very highly expressed in the brain and central nervous system. The editing of *Gria2* at the Q/R site is ADAR2 specific and is required to recode the transcript to form a functional GluA2 protein and allow survival. ADAR3 competes with ADAR1 or ADAR2 for binding to dsRNA substrates, which then are protected from editing due to ADAR3 not having deamination activity

These genetics studies have refined our understanding of the functions of A-to-I editing and of the individual roles that ADAR isoforms fulfill in vivo: ADAR2 is key to site-selective editing, especially in the CNS, whereas ADAR1-mediated editing has an essential role in the prevention of activation of the cytosolic dsRNA innate immune sensing system by endogenous RNA (Fig. 1).

## Dynamic regulation of editing

While our appreciation of the numbers and extent of editing has rapidly expanded, it is less well understood how this process is physiologically regulated. For example, it is established that the same RNA transcript in different regions of the brain is subjected to variable levels of editing [28, 31]. Studies have now described A-to-I editing from very early development in single cells to the analysis of a specific brain region over a cohort spanning a large proportion of the lifespan of humans [107, 108]. Such studies have identified the dynamic regulation of A-to-I editing, both temporally and developmentally, indicating a process modulated at multiple levels.

A key contributor to the difference is the distinct patterns of expression of the ADAR proteins. Our recent work analysing thousands of human RNA-seq data sets from the GTEx project revealed that the expression of ADARs partially, but not fully, accounts for the variation of RNA editing levels [76]. Different ADARs appear to play distinct roles. Specifically, ADAR1 and ADAR2 expression can explain about 20 and 2.8%, respectively, of the variation in overall editing of repetitive sites. In constrast, for non-repetitive protein-coding sites, ADAR1 and ADAR2 expression can explain 6 and 25% of the variation, respectively. Intriguingly, ADAR3, which is enzymatically inactive, negatively affects RNA editing, possibly by competing with ADAR1 and ADAR2 to bind the editing substrates, a finding consistent with observations in model organisms [76]. These findings suggested important roles of ADARs in regulating RNA editing, but also prompt searches for additional regulators and modifiers of RNA editing to better account for the editing variation. These include the influence of the structure of the dsRNA containing the targeted adenosine, the neighboring bases to the editing site and the influence of other RNA binding proteins or modifers of ADAR function. Collectively these factors combine to result in the observed level of editing for a given site.

## *Cis* regulation of A-to-I RNA editing

**Fig. 2:**
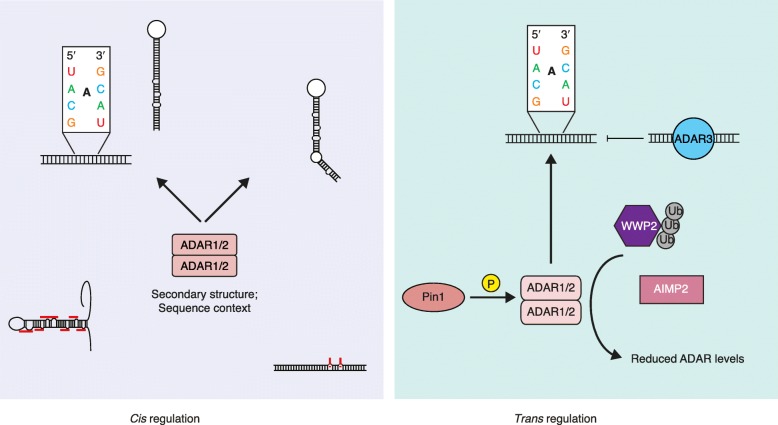
*Cis* versus *trans* regulation of A-to-I editing. *Cis* regulation contributes significantly to the efficiency of editing by ADARs. The sequence context and secondary structure surrounding the adenosine are important in determining the efficiency of editing. The 5′ and 3′ nucleotides adjacent to the adenosine are important contributors to the editing efficiency. *Trans* regulation contributes less significantly to the overall editing, and can either enhance editing, such as occurs with Pin1 phosphorylation of ADAR, or reduce overall editing, as occurs with WWP2 (ubiquitination of ADAR) or AIMP2 (reduces overall levels of ADAR1)

Both *cis* and *trans* effects contribute to the regulation of RNA editing. *Cis* regulation refers to the primary RNA sequence and secondary dsRNA structure as the substrate for editing. *Trans* regulation indicates that *trans*-acting factors, such as ADARs and other regulators, alter the editing efficiency observed at a given locus (Fig. 2). We have recently generated two independent lines of evidence suggesting that RNA editing is mainly regulated in *cis*. First, when we compare RNA editing of conserved sites in multiple tissues from human, primate, and mouse, the samples are clustered by species types, rather than by tissue types [76]. This is very similar to the findings that RNA splicing regulation is also mainly *cis* directed [109, 110]. Second, using closely related *Drosophila* species, *D. melanogaster* and *D. sechellia*, and their F1 hybrids, we differentiated the effects of *cis* sequences from *trans* regulators by comparing species-specific editing levels in F1 hybrids and their parents. We found that *cis* sequence differences are largely responsible for editing level differences between these two *Drosophila* species, whereas *trans* regulators are likely only responsible for subtle changes [111]. These data prompt us to better understand the underlying rules of RNA editing *cis* regulation.

How ADARs target a specific A-to-I RNA editing site is a long-standing question that is not well addressed. Both the primary sequence and secondary structure (i.e., *cis*-acting regulatory elements) surrounding the editing site guide the preference and selectivity of ADARs. ADAR has a preferred sequence motif neighboring the targeted adenosine, in particular the 5′ and 3′ nearest neighboring positions to the editing site, with the depletion and enrichment of G upstream and downstream of the editing site, respectively [50, 112, 113]. Recent analysis of crystal structures of human ADAR2 deaminase domain bound to substrate RNA now provide a basis for the nearest neighbor preference of ADARs [114]. These structures demonstrated the 5′-neighbor preference for a U or A, as when this base is a G or C there is a destabilizing interaction with the backbone of the ADAR protein which reduces, but does not abolish, the interaction and thus impacts on editing efficiency. Additionally, adenosines edited in a dsRNA are affected by mismatches, bulges, and loops both positively and negatively, implicating complex structural contributions to editing specificity [112, 115]. While these specific examples are informative, they prompt systematic studies to more completely decipher the *cis* regulatory code of RNA editing.

We and others recently applied a quantitative trait locus (QTL) mapping approach to identify genetic variants associated with variability in RNA editing [116–118]. With accurate measurement of RNA editing levels at 789 sites in 131 *D. melanogaster* strains, we identified 545 editing QTLs (edQTLs) associated with differences in RNA editing [117]. We demonstrated that many edQTLs can act through changes in the local secondary structure for edited dsRNAs. Furthermore, we found that edQTLs located outside of the edited dsRNA duplex are enriched in secondary structure [117]. While these studies are unprecedented, future studies are needed to systematically understand the features of RNA sequence and structure to enable deciphering of the *cis* regulatory code of RNA editing. Consistent with these results, an assessment of editing across 21 diverse organisms concluded that editing is enriched in regions of putative double-strandedness and is relatively rare in coding regions [42]. This analysis further confirmed the near unique requirement for editing in cephalopods (octopus/squid), where there is a profoundly elevated level of A-to-I editing [42, 56, 60].

An additional finding from the analysis of the crystal structures of the human ADAR2 deaminase domain bound to an RNA substrate was that differences between the ADAR proteins themselves may affect substrate specificity [114]. It was identified that both ADAR2 and ADAR1 share homology for a previously unrecognized side chain (R510 in ADAR2) which is absent in ADAR3. This residue interacts with the RNA substrate and mutation of the R510 residue in hADAR2 to either a glutamine or an alanine reduced the deaminase activity by an order of magnitude [114]. This difference may be an important contributor to the inability of ADAR3 to edit. It was also reported that there are differences between the RNA-binding loops of ADAR2 and ADAR1. These differences may be important in substrate selection and editing efficiency of a given substrate by ADAR1 and ADAR2. Therefore, the collective effect of RNA substrate structure, the sequence context surrounding the adenosine, and which ADAR protein binds all contribute to the efficiency of editing at a given adenosine.

## *Trans* regulators and modifiers of ADARs and editing efficiency

Beyond ADAR editing enzymes themselves only a handful of proteins have been identified that modulate RNA editing, despite speculation about the existence of additional *trans* regulators involved in the RNA editing machinery. In *Drosophila*, the fragile X protein FMR1 biochemically and genetically interacts with ADAR to influence editing levels [119], the RNA helicase maleless controls the editing of one transcript through regulating its splicing [120], and the transcription factor period is thought to modulate editing at a small number of sites through an unknown mechanism [121]. However, these regulators combined explain editing level regulation at fewer than 1% of known editing sites in *Drosophila*, underscoring the need for additional efforts to identify editing regulators with broader effects.

In mammals, two proteins are known to regulate ADAR2’s global activity through post-translational modifications. Pin1 promotes editing by binding ADAR2 in a phosphorylation-dependent manner, while WWP2 decreases editing by targeting ADAR2 for ubiquitination [122]. By taking advange of the large GTEx dataset, we recently identified AIMP2 as a novel negative regulator of RNA editing because its expression is negatively correlated with overall editing levels across thousands of samples. Further experimental validation demonstrated that AIMP2 acts to inhibit RNA editing, at least partially, through lowering the protein level of ADARs [76]. Additionally, a genetic screen in yeast expressing mammalian ADAR2 identified a handful of mammalian enhancers and suppressors of ADAR2 editing, mostly RNA binding proteins, which appear to regulate a small number of sites [123, 124]. There is a clear need for systematic searches of novel RNA editing regulators in mammals to better explain the dynamic regulation patterns that have been observed.

## ADARs, editing, and disease: what happens when editing goes awry?

The available data suggest a more pronounced separation of biological function between ADAR1 and ADAR2 than was previously expected. Mutations in *ADAR2* have not been reported to be associated with human disease. In contrast, mutations of *ADAR* are associated with the human diseases dyschromatosis symmetrica hereditaria (DSH) [125, 126] and Aicardi–Goutières syndrome (AGS) [127–129]. Over 100 heterozygous *ADAR* mutations have been reported in DSH and are associated with altered pigmentation (areas of hypo- and hyperpigmentation) on the face and dorsal aspects of the extremities that first appear in infancy/early childhood. This condition is not fatal and the symptoms appear to be largely restricted to the skin.

More recently, and contrasting with the phenotypes of DSH, Crow, Rice, and colleagues identified biallelic *ADAR* mutations as one of the genetic causes of AGS [127]. AGS has some clinical features that are similar to congenital viral infections. AGS patients, including those with *ADAR* mutations, develop a severe neurodevelopmental disorder characterized by intracranial calcifications and motor disorders, and have evidence of an activated innate immune/interferon response (“interferonopathy”) in their peripheral blood, consistent with the results from murine mutants [130]. Mutations in eight genes are associated with AGS, with a clustering of genes involved in cytosolic DNA metabolism (*TREX1*, *RNASEH2B*, *RNASEH2C*, *RNASEH2A*, *SAMHD1*) and those regulating cytosolic RNA metabolism (*ADAR* and *IFIH1*) [129, 131]. In AGS, unlike DSH, biallelic mutations of *ADAR* are seen in affected patients and are predicted to be significantly more detrimental to the RNA editing/interacting potential of the mutant proteins. Interestingly, despite the significantly different numbers of repetitive elements between the species (*Alu* repeats are primate restricted), the transcriptional response to ADAR1 deficiency is conserved between mouse and human, as is the specific requirement for MDA5 in this response [78]. These results, corroborated by evidence from murine models, demonstrate that significant reductions in the activity of ADAR1 are poorly tolerated in vivo. In contrast to the deleterious consequences of reduced ADAR1 function in human kindreds, germline mutations in *ADAR2* or *ADAR3* have not yet been clearly described or associated with human disease.

A range of different human diseases are associated with altered editing and ADAR activity. In these cases, the direct mutation of the ADAR genes does not cause this association, as is seen in AGS. There is a significant body of work demonstrating reductions in editing, principally ascribed to ADAR2, in a range of neuronal and CNS disorders, including Alzheimer’s disease and amyotrophic lateral sclerosis [132–136]. In the majority of cases, these studies have reported reduced editing of specific targets in these disease settings when compared with normal tissue or non-affected samples. To date there has not been a clear association of reduced ADAR1 function with diseases of the CNS, outside of the germline diseases noted above. This contrasts with the clinical phenotypes of AGS, when profound changes in the CNS are observed in patients with biallelic mutation in *ADAR*.

Our appreciation of the extent and characteristics of A-to-I editing have rapidly expanded, paralleling the technological advancements in sequencing methods. This has been particularly informative in the context of cancer, where large datasets from diverse human cancers have been harnessed to identify links between altered A-to-I editing levels and a range of different cancer types. Initial reports described changes, generally reductions, of ADAR2-mediated editing at selected targets in tumors of the CNS such as glioblastoma and astrocytoma [137, 138]. Recent studies utilizing large RNA-seq datasets from human cancers have identified a trend of increased overall editing and ADAR1 expression in cancer types ranging from leukemias to solid tumors [33, 95, 139–145]. Reasons for the increased ADAR1 expression have been associated with both copy number gains at chromosome 1, where the *ADAR* gene resides, and the activation of interferon/innate immune sensing responses in tumors leading to an increase in ADAR1 expression. The biological consequences of increased ADAR1 and an increased level of overall editing in tumors is only beginning to be explored. In some specific examples, such as in melanoma, reduced editing efficiency has been proposed to be important in the pathogenesis of these tumors [146, 147], although this appears to be less common than increased expression of ADAR1 and higher overall editing levels. Our understanding of the consequences of changes in A-to-I editing on cancer initiation and maintenance, both at the level of its effect on specific transcripts and also on the global transcriptomome of the cancer cells, is only beginning to be explored, and how this contributes to tumor evolution requires further study.

## Future directions

Our understanding of the landscape of A-to-I editing has rapidly expanded over the past decade. The efforts of many investigators have enabled us to catalogue editing across the transcriptomes of many species. The ability to identify editing with high confidence at the genome scale has enabled a better understanding of how editing contributes to genome diversity in a range of contexts: evolutionarily, developmentally, and pathogenically. Paralleling the identification of A-to-I editing events, studies using genetically modified organisms have greatly enhanced our understanding of the in vivo roles and functions of ADARs. These studies have established that ADAR1 serves a unique function in the regulation of the innate immune response to self-RNA, while ADAR2 principally contributes to editing in a more site-selective manner, and ADAR3 competes with ADAR1/2 for substrates, but does not edit them directly. Further studies have broadened our understanding of factors contributing to A-to-I editing efficiency of a given substrate, principally the *cis* regulation of RNA sequence and structure surrounding the edited adenosine and, to a lesser extent, the *trans* regulation of ADAR protein activity/levels by other cellular proteins. At the cellular level, how altered A-to-I editing, both increased and decreased, impacts cell fate is only beginning to be explored. This is particularly relevant in disease contexts, where evidence has solidified that there is altered activity of ADAR proteins. In inherited disorders such as AGS the loss/reduction of ADAR1 activity has a profound impact on normal functioning and is ultimately lethal. In cancer, where elevated ADAR1 expression and activity have been frequently reported, it remains to be shown if these reflect a function in driving tumor initiation and maintenance or reflect the physiological function of ADAR1, to edit endogenous dsRNA to prevent activation of the innate immune system. Many of the tools developed to allow our present understanding of the physiological roles of ADARs can be applied to understand these pathogenic roles.

Modifications of RNA, outside of A-to-I editing, are increasingly being defined as key regulators of transcriptional output and more than 100 distinct types of modifications have been identified to date [1, 2]. This raises many important questions about how these modifications are co-ordinated and interact with/influence each other, ultimately impacting the fate of the given RNA and cell. Such conceptual models have been established and experimentally defined for the interactions of modifcations impacting DNA and chromatin. As an example of an RNA modification, *N*(6)-methyladenosine (m^6^A) is the most frequent internal modification of mRNA [148]. There are many parallels between the roles identified for m^6^A and those of A-to-I editing, including roles in the viral life cycle [149, 150] and in the regulation of cell fate determination [151–153] and cancer [154–156]. Given their respective prevalence across the transcriptome, how m^6^A and A-to-I editing interact and alter the fate of the targeted RNA transcripts is at present unclear [157]. It may be that these are distinct epitranscriptomic processes that individually impact the fate of a given RNA, or that there is a level of interaction that occurs between these highly prevelant modifications. This will be relevant to normal cell function but also in pathogenic settings. As we understand more about the biological functions of the distinct modifications and the cell types that co-express the enzymes capable of writing, reading and erasing these marks, we will begin to understand the cartography of RNA modifications and how they reshape transcriptome output.

## Abbreviations

ADAR: Adenosine deaminanse acting on RNA
AGS: Aicardi–Goutières syndrome
CNS: Central nervous system
DSH: Dyschromatosis symmetrica hereditaria
dsRNA: Double-stranded RNA
edQTL: Editing quantitative trait locus
QTL: Quantitative trait locus
